# *Arih2* gene influences immune response and tissue development in chicken

**DOI:** 10.1042/BSR20190933

**Published:** 2019-10-18

**Authors:** Guanxian Wu, Sifan Xu, Wanting Zhang, Yang Liu, Qiuyuan Wang, Chaolai Man

**Affiliations:** College of Life Science and Technology, Harbin Normal University, Harbin 150001, P.R. China

**Keywords:** Arih2, chicken, clone, expression, immune

## Abstract

Ariadne homolog 2 (ARIH2), as an E3 ubiquitin ligase, is one of the important factors involved in regulating biological functions, such as inflammation and skeletal muscle degeneration. In the present study, the full-length coding sequence of *Arih2* gene was cloned from Hy-Line Brown chicken. The tissue transcriptional profiles of *Arih2* gene at different developmental stages were detected using quantitative real-time PCR (qRT-PCR), and the *Arih2* functional characteristics in immune response were analyzed. The results showed that the full-length coding sequence of *Arih2* gene was 1473 bp, encoding 490 amino acids, and conservative between different species. The *Arih2* gene was transcribed in various tissues at different developmental stages, and its transcriptional activities varied significantly between multiple tissues. With the development of chicken, *Arih2* gene was basically up-regulated in heart, liver, kidney, skeletal muscle and glandular stomach, but fluctuated significantly in large intestine. In immune response, the transcriptional activities of *Arih2* gene exhibited significant changes in the bursa, thymus and blood (*P*<0.05). The results showed that *Arih2* might be a multifunctional gene involved in tissue development and immune response in chicken, and have a potential possible application as diagnostic marker for identifying immune response.

Ariadne homolog 2 (ARIH2, also known as TRIAD1) belongs to a RING1-in-between-RING2 (RBR) E3 ligase family, which is a type of E3 ubiquitin ligase with RING domain and synthesis mechanism of ubiquitin chain, and plays important physiological functions in nucleus [1,2]. ARIH2 can coordinate aging-associated skeletal muscle degeneration with the muscle regulator polyadenylate-binging protein nuclear 1 (PABPN1) in mouse, and modify skeletal muscle by interacting with TsUBE2L3 in nematode [3–5]. In addition, conditional regulation of *Arih2* transcription by the tyrosine phosphorylation states of HoxA9 and HoxA10 can contribute to terminating the innate immune response in mouse [6]. ARIH2 can antagonize NF-κB signaling by promoting the degradation of nuclear IκBβ, which can activate the NF-κB pathway and promote the occurrence of inflammatory response [1,7]. Also, deletion of endogenous *Arih2* inhibits nucleotide-binding oligomerization domain-like receptor family pyrin domain containing 3 (NLRP3) ubiquitination and promotes NLRP3 inflammasome activation, whereas *Arih2* overexpression promotes NLRP3 ubiquitination and inhibits its activation [8,9]. Moreover, study has confirmed that *Arih2* gene is associated with inflammatory bowel disease and can become a potential therapeutic target for inflammatory diseases [10].

Since ARIH2 plays key roles in the immune response [1,6], inflammation [1,8,12] and skeletal muscle modification [4,5], it is necessary to study the functions and characteristics of chicken *Arih2* gene. At present, inflammation-related diseases and growth rates are still main problems affecting poultry industry. Studying chicken *Arih2* gene may provide a positive reference for solving these problems. In addition, related studies on *Arih2* gene have not been reported in chicken. In the present study, the full-length coding sequence of chicken *Arih2* was cloned from Hy-Line Brown chicken and analyzed. Then, the *Arih2* transcriptional profiles at different developmental stages were analyzed. Finally, the possible functions of *Arih2* were explored in immune response. The present study can lay the foundation for further studying *Arih2* functions.

## Materials and methods

### Experimental animal and tissue collection

The Hy-Line Brown chickens (14-day-old, 10- and 24-month-old) were obtained from Northeast Agricultural University poultry farm. The 16 tissues (heart, liver, spleen, lung, kidney, brain, skeletal muscle, muscle stomach, thymus, skin, small intestine, large intestine, glandular stomach, fat, blood and bursa) were separated from the three different ages of chickens respectively, then frozen in liquid nitrogen and stored at −80°C.

### Cloning and bioinformatics analysis of *Arih2* gene

Total RNA from heart, liver, spleen, lung, kidney, brain, skeletal muscle, thymus, muscle stomach, skin, small intestine, large intestine, glandular stomach, fat, bursa and blood of chickens at the three different ages were extracted using TRIzol reagent (Sigma–Aldrich, St. Louis, MO, U.S.A.) and reverse transcribed into cDNA using FSQ-301 kit (TOYOBO, Shanghai) according to the manuals, respectively. The coding sequence (CDS) of *Arih2* gene was amplified by PCR with primers based on the predicted nucleotide sequence of *Arih2* from *Gallus gallus* (GenBank: NM_001199221), and the muscle stomach cDNA of 14-day-old chicken as a template. The primer sequences were as follows: 5′-GCTCAGATGAAATCAAAGGAACGGT-3′ and 5′-CTGCTTATCCCGGTGAAGCTGCCAT-3′. The total PCR system was 25 μl including: 0.5 μl cDNA (20 ng/μl), 0.8 μl of each primer, 2 μl dNTP mixture (2.5 mM), 2.5 μl of 10× ExTaq buffer, 1 μl ExTaq™ DNA polymerase (TaKaRa Bio Inc.) and 17.4 μl sterile deionized water. The PCR procedure was as follows: 95°C/5 min; 30 cycles of 94°C/1 min, 65°C/1 min, 72°C/45 s; 72°C/10 min; 4°C to terminate the reaction. The PCR products were cloned into vector PMD18-T (TaKaRa, Dalian) and identified by double digestion with restriction enzymes (*Bam*H I and *Sal* I). Five positive clones with the correct identification were randomly selected and sequenced by Shanghai Bioengineering Co., Ltd. The nucleotide and amino acid sequences of *Arih2* gene were analyzed using SOPMA, DNAMAN and MEGA7.0 software etc.

### Transcriptional profile analysis of *Arih2* gene

Quantitative real-time PCR (qRT-PCR) was used to analyze the transcriptional activities of *Arih2* gene in each tissue. The qRT-PCR system doses and reaction procedures of the *Arih2* gene were the same as the internal reference β*-actin* (GenBank number: NM205518). The primers used for qRT-PCR were as follows: target gene *Arih2*: 5′-AGGGACTATGTGGAGAGCCATTACC-3′ and 5′-AAGCAGAAGACCTCATTGCAACGAT-3′; internal reference gene β*-actin*: 5′-CACCAACTGGGATGATAT-3′ and 5′-CGTACTCCTGCTTGCTGATC-3′. qRT-PCR total system was 20 μl including: 10 μl 1× SYBR Green I (TOYOBO, Shanghai), 0.4 μl 50× ROX reference dye (TOYOBO, Shanghai), 0.6 μl of each primer, 2 μl cDNA and 6.4 μl ddH_2_O. The reaction procedure was as follows: 95°C/1 min; 40 cycles of 95°C/15 s, 56°C/30 s, 72°C/40 s.

### Transcriptional characteristics of *Arih2* gene in immune response

In order to verify whether *Arih2* was involved in immune response, the 11-day-old Hy-Line Brown chickens were vaccinated with LaSota (Heilongjiang Biological Production Company, Harbin, China) according to the manufacturer’s instructions by the eye-drop method [11]. Five immune-related tissues such as blood, liver, spleen, thymus and bursa were collected on the day 14 post-vaccination. The methods of total RNA extraction, reverse transcription and qRT-PCR were the same as that in ‘Transcriptional profile analysis of Arih2 gene’ section.

### Statistical analysis

In the present study, three chickens were randomly selected for qRT-PCR analysis, and each chicken was subjected to three technical repetitions. The relative transcriptional activity of *Arih2* was analyzed using 2^−Δ*C*_t_^ method. The data were analyzed by one-way ANOVA using SPSS 20.0 software, and finally plotted using GraphPad Prism7.0 software.

## Results and discussion

ARIH2 has been shown to be widely involved in important physiological processes, such as innate immune response and inflammation [1,6,8,12]. In this study, the *Arih2* gene was cloned and its transcriptional profiles were analyzed at different developmental stages. In order to understand the possible functions of *Arih2* in adaptive immune response, the transcriptional activities of *Arih2* mRNA in immune-related tissues were identified in immunized chicken.

**Figure 1:**
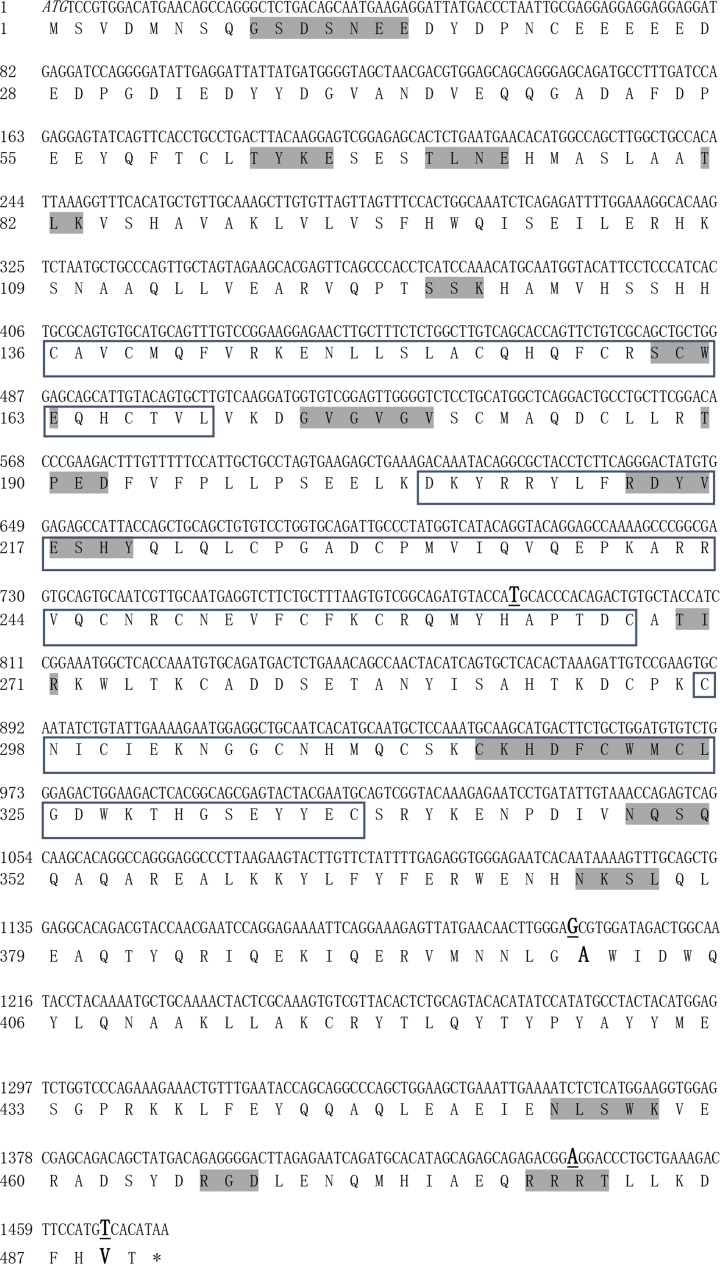
The complete coding sequence of Hy-Line Brown chicken *Arih2* gene and its encoding amino acids The italics indicate the start codon; the star (*) indicates the stop codon; the bold and underline indicate the SNP sites, and the bold indicates the amino acid mutation due to the base mutation; the sequences in the box represent conserved domain (RING1: 136-169AA, IBR: 205-267AA; RING2: 297-337AA). Functional sites are shaded (N-glycosylation site (348-NQSQ-351, 373-NKSL-376 and 453-NLSW-456), cAMP- and cGMP-dependent protein kinase phosphorylation sites (479-RRRT-482), Protein kinase C phosphorylation site (63-TYK-65, 81-TLK-83, 124-SSK-126, 269-TIR-271 and 455-SWK-457), Casein kinase II phosphorylation site (12-SNEE-15, 63-TYKE- 66, 70-TLNE-73, 160-SCWE-163 and 189-TPED-192), Tyrosine kinase phosphorylation site (213-RDYVESHY-220), N-myristoylation site (9-GSDSNE-14 and 173-GVGVGV-178), Cell attachment sequence (466-RGD-468), Zinc finger RING-type signature (315-CKHDFCWMCL- 324)).

**Figure 2:**
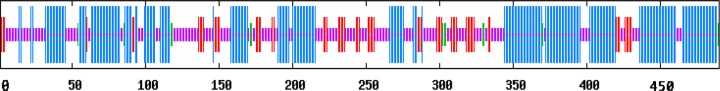
The secondary structure of chicken ARIH2 protein The result was predicted by the SOPMA (https://npsa-prabi.ibcp.fr/cgi-bin/npsa_automat.pl?page=npsa_sopma.html) software, and the configurations represented by the length of the line segment from long to short are α-helix, extended chain, β-turn and random coil. The numbers represent the location of amino acids. RING1-IBR-RING2 (136-337AA).

**Figure 3:**
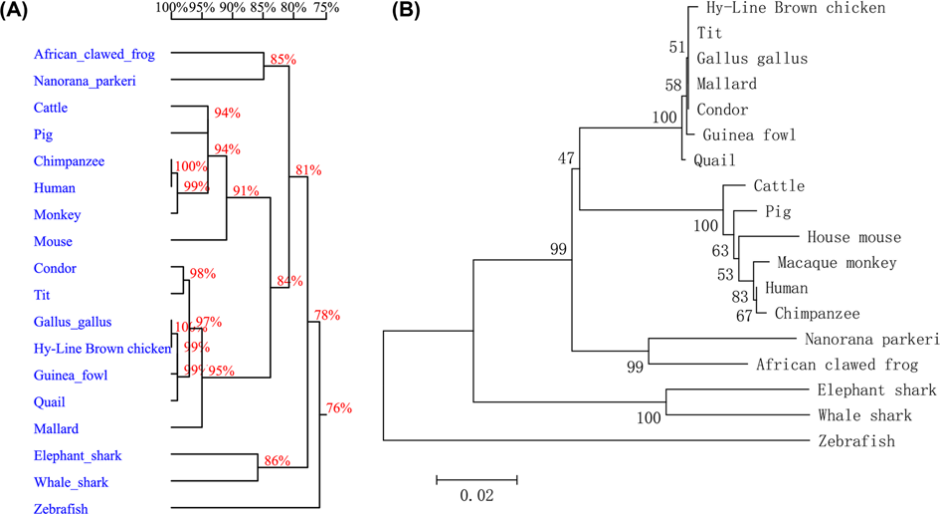
Homology and evolution analysis of multiple sequences of *Arih2* gene (**A**) Nucleotide homology analysis of *Arih2* coding sequences using DNAMAN software (http://www.Lynnon.com). Interval range of scale corresponds to homology value of homologous tree. Percentages on branches denote similarity between sequences. (**B**) Phylogenetic tree analysis of ARIH2 amino acid sequences. The phylogenetic tree was obtained by boot strap analysis with the neighbor-joining method. Numbers on the branches represent bootstrap values for 1000 replications. Scales represent indicating differences between display sequences, and 0.02 indicates that two of the 100 bases are different, and the branching value represents its bootstrap value. Human (GenBank:NM_001317333.1); Chimpanzee (GenBank:NM_001280136.1); Mouse (GenBank:NM_001357283.1); Cattle (GenBank:NM_001206242.2); Pig (GenBank:XM_003132197.6); Macaque monkey (GenBank:XM_015130961.1); *Gallus gallu*s (GenBank:NM_001199221); Hy-Line Brown chicken (GenBank:MK_335707); Guinea fowl (GenBank:XM_021409574.1); Quail (GenBank:XM_015875271); African clawed frog (GenBank:NM_001096776); Whale shark (GenBank:XM_020527432.1); Condor (GenBank:XM_010571148.1); Mallard (GenBank: XM_027468046.1); *Nanorana parkeri* (GenBank:XM_018576133.1); Zebrafish (GenBank:NM_213143.1); Elephant shark (GenBank:XM_007890321.1); Tit (GenBank:XM_015640917.2).

The sequencing results showed that the coding region of *Arih2* gene was 1473 bp and encoded 490 amino acids. Based on the predicted *Arih2* gene of *Gallus gallus, Arih2* gene was located on chromosome 12 with sixteen introns and seventeen exons, and there were four SNPs in the coding region of Hy-Line Brown chickens *Arih2* gene: the mutation points were C to T (position 786 nt), A to G (position 1198 nt) which resulted in the mutation of amino acid (position 400 AA) from threonine (T) to alanine (A), C to A (position 1441 nt), A to T (position 1466 nt) which resulted in the mutation of amino acid (position 489 AA) aspartic acid (D) to proline (V) (Figure 1). Multiple glycosylation and phosphorylation functional sites (N-glycosylation site, cAMP- and cGMP-dependent protein kinase phosphorylation site, Protein kinase C phosphorylation site and a cell attachment site) were located in the C-terminus of ARIH2 protein, which suggested that the ARIH2 C-terminal might play an important role in the cellular localization and protein modification. Structural analysis showed that chicken ARIH2 protein had a secondary structure dominated with the C-terminal α-helixes and a symbolic structure domain (RING1-IBR-RING2) of the ariadne RBR family (Figures 1 and 2). In addition, homology comparison and evolutionary analysis were carried out based on the sequences of nucleotide and amino acid of *Arih2* gene coding regions among 18 species, respectively (Figure 3). The results showed that the *Arih2* genes had high homology between different species, and chicken ARIH2 was in the middle position of the higher mammal and the amphibian and fish in evolution, which suggested that chicken was an excellent model animal for studying *Arih2* gene.

**Figure 4:**
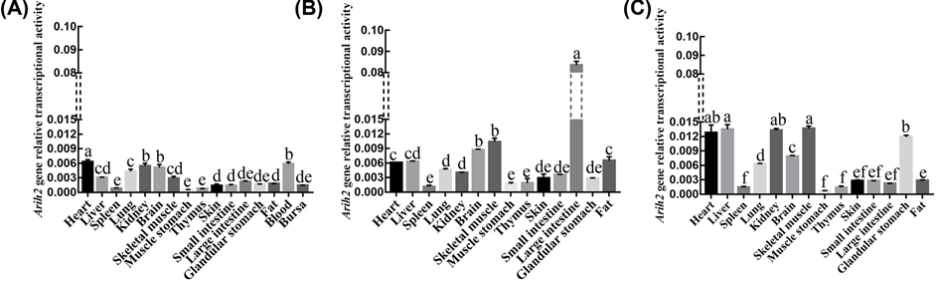
Transcriptional levels of *Arih2* gene in different developmental stages of Hy-Line Brown chicken (**A**) Relative transcriptional activity analysis of *Arih2* gene in 14-day-old Hy-Line Brown chicken. (**B**) Relative transcriptional activity analysis of *Arih2* gene in 10-month-old Hy-Line Brown chicken. (**C**) Relative transcriptional activity analysis of *Arih2* gene in 24-month-old Hy-Line Brown chicken. The letters on bars in the figure represent significant differences in transcriptional activity between tissues. The data are indicated as mean ± SD. Different letters (a–f) represent significant differences (*P*<0.05), while the same letters represent no significant differences (*P*>0.05).

Expression profiling analysis revealed that chicken *Arih2* mRNA was transcribed in all tissues, but the transcriptional activities had differences in the same tissues at different development stages or in the different tissues at the same age (Figure 4). According to the range of relative transcriptional activity, we divided it into three levels. For example, in 14-day-old chickens, *Arih2* was highly transcribed (the relative transcriptional activity value was higher than 0.006) in heart, medium transcriptional activity (the value of relative transcriptional activity was between 0.003 and 0.006) in the liver, lung, kidneys and brain, and low transcriptional activity (the value of relative transcriptional activity was below 0.003) in the spleen, muscle, stomach, small intestine, large intestine, skin, thymus, bursa and fat. In 10-month-old chickens, *Arih2* was highly transcribed in the heart, liver, brain, muscle, large intestine and fat, medium transcriptional activity in the lung, kidney and small intestine, and low transcriptional activity in the other tissues; In 24-month-old chickens, *Arih2* was highly transcribed in the heart, liver, lungs, glandular stomach, kidneys and muscle, and low transcriptional activity in the rest of tissues. Basically, with different stages of development, the transcriptional activity of *Arih2* gene was up-regulated in many tissues (such as heart, liver, kidney, lung, brain, skeletal muscle and glandular stomach), but there was no corresponding change in a few tissues (spleen, thymus, skin, muscle stomach, fat and small intestine), suggesting that *Arih2* gene might have different functions in different tissues. Studies have shown that ARIH2 regulates PABPN1 protein turnover and affects muscle cell fusion, and ARIH2 can suppress ubiquitination and degradation of skeletal muscle-specific proteins through binding TsUBE2L3, thus make muscle proteins more stable [3–5]. In this study, *Arih2* was highly transcribed in heart and skeletal muscle of the three different developmental stages, which suggested that *Arih2* might be involved in the positive regulation of the muscle development. Interestingly, *Arih2* transcription was significantly up-regulated in the large intestine tissue of 10-month-old chickens. Studies showed that E3 ubiquitin ligases ITCH, ring finger protein 4 (RNF4) and ubiquitin fold modifier 1 (Ufm1) played important roles in maintaining intestinal homeostasis and mucosal immunity [13–15]. *Arih2*, as E3 ubiquitin ligase gene, had high transcriptional activity in the large intestine of 10-month-old chickens that were in the stage of rapid growth and enhanced digestibility. It was speculated that *Arih2* might also participate in the proliferation and formation of defense barrier of large intestinal epithelial cells, thus maintaining the stability of the intestinal environment.

**Figure 5:**
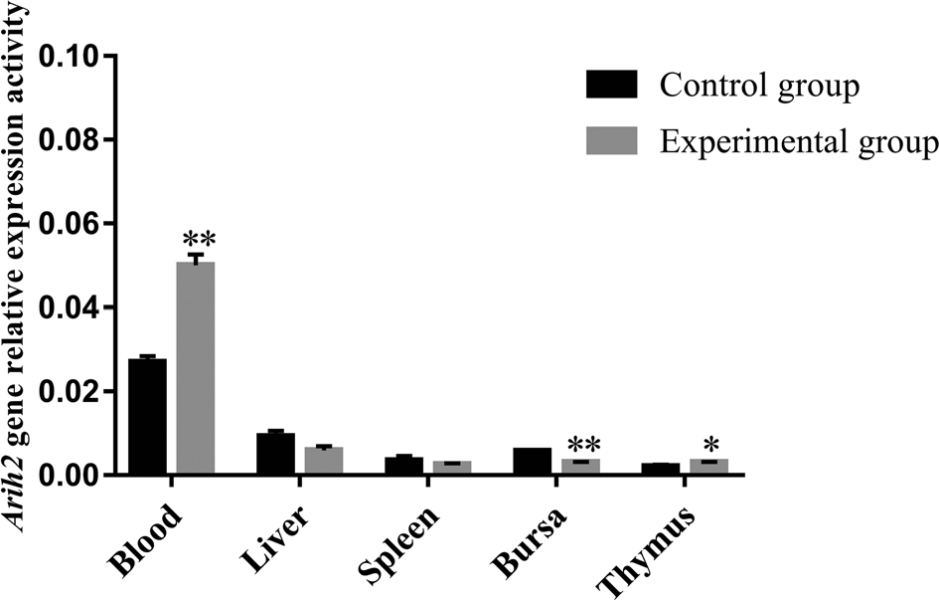
Expression changes in *Arih2* gene on the day 14 post-vaccination The 11-day-old Hy-Line Brown chickens of experimental group were vaccinated with LaSota, while age-matched chickens in the control group were administered with PBS (20 mM phosphate, 150 mM NaCl, pH 7.4) by the same method. The data are indicated as mean ± SD. The * and ** represent the significant difference between the experimental group and control group. * indicates statistically significant (*P*<0.05) and ** indicates statistically highly significant (*P*<0.01).

Previous study in our lab showed that the titers of antibodies were in the ascending stage at 14 days after vaccine immunization, which was the positive reaction period of acquired immune response and could better reflect the expression status and functional characteristics of target genes [11]. The results of transcriptional activity analysis showed that *Arih2* was significantly up-regulated in the blood of immunized chickens (Figure 5). Erythrocytes are the most abundant blood cells in the blood and play an important role in the body’s anti-infective immunity. Study showed that human HB (hHB) could enhance the RIG-I-mediated antiviral responses through promoting the RIG-I ubiquitination depending on the hHB-induced reactive oxygen species (ROS) [16]. Therefore, it was speculated that the high transcriptional activity of *Arih2* in blood might mainly come from erythrocytes, and ARIH2, an E3 ligase, might be involved the RIG-I ubiquitination process and participate in the anti-infective immune function of erythrocytes. In addition, chicken blood can be obtained easily, and several studies have shown that the changes of target gene mRNA levels can be used as diagnostic markers or detection indicators for tumors, cancers and myelodysplastic diseases [17–19], so the significant changes of *Arih2* gene transcriptional activity in blood might become a potential diagnostic marker for identifying immune response, which might be helpful for serological antibody detection. Moreover, the transcriptional activity changes of *Arih2* in the central immune organs (bursa and thymus) were opposite and different obviously. The *Arih2* transcriptional activity was distinctly up-regulated in the thymus, but significantly down-regulated in the bursa. However, there were no significant transcriptional differences between the peripheral immune organs (liver and spleen) (Figure 5). Study showed that if *Arih2* was absent from mice, T-cell tissue penetration would occur and then stimulated immune response [1]. In the central immune organs, the significant changes of *Arih2* suggested that *Arih2* might be highly correlated with the differentiation and maturation of T and B cells, but the participation mechanisms might be different. The down-regulation of *Arih2* transcripts in liver and spleen further enhanced immune response, because ARIH2 expression activity was negatively correlated with immune response [1,6].

In conclusion, we first cloned chicken *Arih2* gene and analyzed transcriptional profiles. With the development of chicken, the transcriptional activity of *Arih2* increased gradually in multiple tissues, and fluctuated significantly in large intestine. The transcriptional activity of *Arih2* gene was up-regulated significantly in blood during immune response, which suggested that *Arih2* gene might have a potential application value for identifying immune response. The results implied that *Arih2* gene was involved in tissue development and adaptive immune response. These data can serve as a foundation for further studying *Arih2* gene.

## Abbreviations

ARIH2: Ariadne homolog 2
HB: Hemoglobin
hHB: human HB
HoXA: HomeoboxA
IκBβ: Inhibitor of kappa B-beta
NLRP3: nucleotide-binding oligomerization domain-like receptor family pyrin domain containing 3
PABPN1: polyadenylate-binging protein nuclear 1
qRT-PCR: quantitative real-time PCR
RIG-I: Retinoic acid inducible gene I
